# Regulation by Dietary Carbohydrates of Intermediary Metabolism in Liver and Muscle of Two Isogenic Lines of Rainbow Trout

**DOI:** 10.3389/fphys.2018.01579

**Published:** 2018-11-13

**Authors:** Xuerong Song, Lucie Marandel, Sandrine Skiba-Cassy, Geneviève Corraze, Mathilde Dupont-Nivet, Edwige Quillet, Inge Geurden, Stephane Panserat

**Affiliations:** ^1^UMR1419 Nutrition, Metabolisme, Aquaculture, Aquapôle, E2S UPPA, INRA, Université de Pau et des Pays de l’Adour, Saint-Pée-sur-Nivelle, France; ^2^University of Chinese Academy of Sciences, Beijing, China; ^3^State Key Laboratory of Freshwater Ecology and Biotechnology, Institute of Hydrobiology, Chinese Academy of Sciences, Wuhan, China; ^4^GABI, INRA, AgroParisTech, Université Paris-Saclay, Jouy-en-Josas, France

**Keywords:** glycolysis, gluconeogenesis, fatty acid metabolism, cholesterol metabolism, mitochondrial metabolism, genetic variability

## Abstract

Rainbow trout (*Oncorhynchus mykiss*) is recognized as a typical “glucose-intolerant” fish, and the limits of dietary carbohydrate utilization have been investigated for many years. In this study, the objective was to test the molecular effects of dietary carbohydrates on intermediary metabolism in two major metabolic tissues, liver and muscle. Another objective was also to study if the response to carbohydrate intake depended on the genetic background. We fed two isogenic lines of rainbow trout (named A22h and N38h) with high carbohydrate diet (carbohydrate, 22.9%) or low carbohydrate diet (carbohydrate, 3.6%) for 12 weeks. Carbohydrates were associated with higher feed utilization owned by the well-known protein-sparing effect, with better fish growth performance. However, atypical regulation of glycolysis and gluconeogenesis in liver and absence of *hk* and *glut4* induction in muscle, were also observed. Regarding the effects of carbohydrates on other metabolism, we observed an increased, at a molecular level, of hepatic cholesterol biosynthesis, fatty acid oxidation and mitochondrial energy metabolism. Genetic variability (revealed by the differences between the two isogenic lines) was observed for some metabolic genes especially for those involved in the EPA and DHA biosynthetic capacity. Finally, our study demonstrates that dietary carbohydrate not only affect glucose metabolism but also strongly impact the lipid and energy metabolism in liver and muscle of trout.

## Introduction

Fish liver is the main tissue involved in the regulation of glucose homeostasis (Pilkis and Granner, 1992; Klover and Mooney, 2004). Dietary glucose enters the liver passing through the cellular barrier by using the specific glucose transporter 2, and then it is catabolized through glycolysis pathway for providing ATP, being stored as glycogen or being converted into lipid if in excess (Panserat et al., 2009; Polakof et al., 2011, 2012; Kamalam et al., 2012). To explain the poor use of dietary carbohydrates by rainbow trout, a poor induction of hepatic lipogenesis and an absence of inhibition of hepatic gluconeogenesis have been put forward as major reasons for post-prandial hyperglycemia (Enes et al., 2009; Polakof et al., 2012). Recently, using two specific isogenic trout lines A32h and AB1h, we confirmed that the first steps of glycolysis and gluconeogenesis catalyzed by the glucokinase and the phospenolpyruvate carboxykinase were regulated as expected at the molecular level by dietary carbohydrates; by contrast, surprisingly, some of the genes encoding the last key enzymes involved in glycolysis were down-regulated whereas some of the genes encoding gluconeogenic enzymes were up-regulated (Song et al., 2018). These isogenetic fish are highly considerable for their low inter-individual variability of each genotype (Quillet et al., 2007), whether these results could be observed one more time in standard families remain questionable. Thus, to be confirmed, these unexpected observations related to glucose metabolism (Song et al., 2018) have to be renewed in other fish trout lines, presently the A22h and N38h isogenic lines. In addition, tissues other than liver may be important for glucose use in fish, such as white muscle, the largest tissue in fish. Even though some studies revealed that muscle glycogen was increased by carbohydrate intake (Soengas et al., 1996; Hemre et al., 2002), it seems that there was a low capacity of inducting glycolysis as reflected by low hexokinase activities (Kirchner et al., 2005). Because relative few studies have focused on glucose metabolism in muscle, it is done in the present study using the two isogenic trout lines A22h and N38h.

Dietary glucose is not only a source of energy (catabolism) but is also one of the main metabolic intermediaries which could also affect the synthesis (anabolism) of other biologically important compounds (Wilson, 1994; Polakof et al., 2012). Indeed, carbohydrate-enriched diets can lead to an enhancement of lipogenesis – even at a low level – (Towle et al., 1997; Skiba-Cassy et al., 2009), a decrease of fatty acid oxidation (Randle, 1998), an increase of the long chain polyunsaturated fatty acid (LC-PUFA) which induce synthesis of EPA and DHA (Seiliez et al., 2003; Kamalam et al., 2013). H-CHO also affect hepatic cholesterol metabolism (Kamalam et al., 2012; Deng et al., 2013; Zhu et al., 2017) and modify the energy metabolism in the mitochondria (Panserat et al., 2017). Our objective in the present study is thus to analyze, for the first time, the effect of dietary carbohydrate in these non-glucose metabolic pathways in rainbow trout.

In this context, we fed the two new isogenic trout lines named A22h and N38h with either a L-CHO or a H-CHO for 12 weeks (the same diets used for the A23h and AB1h in our previous study; Song et al., 2018). We analyzed growth performance, whole body composition, plasma metabolites and glycogen contents, with the analysis of the mRNA levels of the key hepatic-muscle enzymes involved in the glucose metabolism including glycolysis and gluconeogenesis, lipid metabolism including lipogenesis, fatty acid oxidation and LC-PUFA biosynthesis, cholesterol metabolism and mitochondrial energy metabolism.

## Materials and Methods

### Ethics Statement

Experimentation was conducted in the INRA experimental facilities (Donzacq, UMR Numéa, St-Pée-sur-Nivelle, France) authorized for animal experimentation by the French veterinary service which is the competent authority (A 64-495-1). The experiments were in strict accordance with EU legal frameworks related to the protection of animals used for scientific research (Directive 2010/63/EU) and according to the National Guidelines for Animal Care of the French Ministry of Research (decree n°2013-118, February 1st, 2013). In agreement with ethical committee “Comité d’éthique Aquitain poissons oiseaux” (C2EA-73), the experiment reported here did not need approval by a specific ethical agreement since it implies only classical rearing practices with all diets used in the experimental formulated to cover all the nutritional requirements of rainbow trout (The National Research Council [NRC], 2011).

### Fish Experimental Diets

Low carbohydrate diet and H-CHO were manufactured at INRA, Donzacq, Landes, France. They were formulated as extruded pellets, the temperature of extruded pellets was 37°C, and the pressures were 46 and 43 bars for L-CHO and H-CHO diets respectively. The two dietscontained different carbohydrates contents but with the same level of proteins and lipids as shown in Table 1. Dietary protein (∼48%) was provided by fishmeal and soluble fish protein concentrate, dietary lipid (8∼11%) was provided by fish oil and fishmeal, and gelatinized starch was included as carbohydrate source because of its higher digestibility (The National Research Council [NRC], 2011) [3.6% (L-CHO) and 22.9% (H-CHO) respectively]. The digestible gelatinized starch in H-CHO diet was compensated by non-digestible cellulose in L-CHO diet (in order to maintain the same level of proteins), which implied a difference in digestible energy content between both diets.

**Table 1:** Formulation and proximate composition of the two experimental diets.

	L-CHO	H-CHO
**Ingredients (%)**		
Fish meal^1^	60.0	60.0
Soluble fish protein concentrate^2^	6.0	6.0
Fish oil^3^	6.0	6.0
Starch^4^	0	25.0
Vitamin premix^5^	1.5	1.5
Mineral premix^6^	1.5	1.5
Cellulose^7^	20.0	0
Wheat^8^	5.0	0
**Proximate composition**		
Crude protein (% DM)	47.7	47.8
Crude lipid (% DM)	11.3	8.1
Gross energy (kJ g^-1^ DM)	20.5	20.8
Carbohydrates (% DM)	3.6	22.9


*L-CHO, low carbohydrate diet; H-CHO, high carbohydrate diet; DM, dry matter. ^*1*^Sopropeche, Boulogne-sur-Mer, France; ^*2*^Sopropeche, Boulogne-sur-Mer, France; ^*3*^Sopropeche, Boulogne-sur-Mer, France; ^*4*^gelatinized corn starch (Roquette, Lestrem, France); ^*5*^supplying (kg^-*1*^ diet): 60 IU _*DL-*α-_tocopherol acetate, 5 mg sodium menadione bisulphate, 15,000 IU retinyl acetate, 3,000 IU _*DL*_-cholecalciferol, 15 mg thiamine, 30 mg riboflavin, 15 mg pyridoxine, 0.05 mg vitamin B12, 175 mg nicotinic acid, 500 mg folic acid, 1000 mg inositol, 2.5 mg biotin, 50 mg calcium panthothenate and 2000 mg choline chloride; ^*6*^supplying (kg^-*1*^ diet): 2.15 g calcium carbonate (40% Ca), 1.24 g magnesium oxide (60% Mg), 0.2 g ferric citrate, 0.4 mg potassium iodide (75% I), 0.4 g zinc sulfate (36% Zn), 0.3 g copper sulfate (25% Cu), 0.3 g manganese sulfate (33% Mn), 5 g dibasic calcium phosphate (20% Ca,18% P), 2 mg cobalt sulfate, 3 mg sodium selenite (30% Se), 0.9 g potassium chloride and 0.4 g sodium chloride; ^*7*^Sopropeche, Boulogne-sur-Mer, France; ^*8*^Sopropeche, Boulogne-sur-Mer, France.*

### Isogenic Fish Lines, Nutritional Experiment and Sampling Procedure

Two heterozygous isogenic lines of rainbow trout (*Oncorhynchus mykiss*) were obtained by mating sires and dams from homozygous isogenic lines (Peima, France). The homozygous lines used as broodstock had been previously established after two generations of gynogenesis and then maintained by within line single pair mating using sex reversed XX males (Quillet et al., 2007). Eggs from fully homozygous females from line B57 were mixed and fertilized in two separated batches with milt from two homozygous sires from A22 and N38 lines to produce heterozygous lines named as A22h and N38h respectively. Therefore, differences between the two lines can be attributed only to genetic differences of paternal origin, whilst all individuals within one line shared the same genotype.

Rainbow trout were reared at 18°C in the INRA experimental facilities at Donzacq, Landes, France under natural photoperiod. During the trial, water dissolved oxygen was 9 mg L^-1^, ammonia < 0.01 mg L^-1^, nitrite < 0.04 mg L^-1^, nitrate was about 17 ppm. The quantity of flow was 0.3^∗^s^-1^ by tank, all water of each tank was changed 8 times each hour, and the water volume was 130 l per tank. Fish of each line (∼5 g) (A22h and N38h) were randomly distributed into tanks at the density of 20 fish/tank, each line being fed with (L-CHO) or without carbohydrates (H-CHO). Fish were reared in triplicates for all the experimental conditions (2 lines ^∗^ 2 diets in triplicate i.e., *n* = 12 tanks in total). All fish were fed twice daily with an interval 8 h to apparent satiation. After 12 weeks of feeding, two fish per tank were randomly sampled at 6 h after the last meal, known to be the peak of posprandial glycemia in rainbow trout fed with carbohydrates at 18°C (Polakof et al., 2012; Kamalam et al., 2017). Trout were anesthetized with benzocaine (30 mg/L) and killed by a sharp blow to the head. Blood was removed from the caudal vein via heparinized syringes and centrifuged (3000 *g*, 5 min). The plasma recovered was immediately frozen and kept at -20°C until analysis. The fresh liver and muscle were collected and immediately frozen in liquid nitrogen and then kept at -80°C. Later, six more fish per tank were randomly sampled at 48 h after the last meal. They were immediately frozen and kept at -20°C for whole body composition determination.

### Chemical Analysis for Diets and Whole-Body Composition

The chemical composition of diets and fish were analyzed by the following procedures: protein content (N × 6.25) was determined by Kjeldahl method after acid digestion; fat was determined by petroleum ether extraction (Soxtherm), gross energy was determined in an adiabatic bomb calorimeter (IKA, Heitersheim Gribheimer, Germany); starch content was determined by an enzymatic method (*in vivo* Labs).

### Metabolites Analysis

Plasma glucose, triglyceride and cholesterol were determined by using commercial kits. Plasma glucose and triglyceride were assayed by the kits from Biomerieux, Marcy I’Etoile, France, and the kit for plasma cholesterol determination was from Sobioda, France. They were adapted to microplate format according to the manufacturer’s instructions. Liver and muscle glycogen was determined by a hydrolysis technique previously described by Good et al. (1993) and Song et al. (2018).

### mRNA Levels Analysis

Liver and muscle samples were extracted using TRIzol reagent (Invitrogen, Carlsbad, CA, United States), according to the manufacturer’s recommendations and were quantified by spectrophotometry (absorbance at 260 nm). The integrity of the samples was assessed using agarose gel electrophoresis. 1 μg of total mRNA per sample was reverse transcribed into cDNA using the SuperScript III reverse transcriptase kit (Invitrogen, Carlsbad, CA, United States) with random primers (Promega, Charbonnieres, France) according to the manufacturer’s instructions.

mRNA levels of the selected genes were determined by quantitative real-time (q) RT-PCR. *ef1α* was regarded as reference gene which was stably expressed in the studies of Olsvik et al. (2005) and the primers sequences of target genes had already been published in previous studies (Marandel et al., 2015, 2016; Liu et al., 2017; Panserat et al., 2017; Zhu et al., 2017) shown in Table 2. The paralogous genes were named according to ZFIN Nomenclature guidelines^1^ by Marandel et al. (2015). In the present study we analyzed the mRNA levels of genes encoding glycolytic enzymes (*gck*, coding the glucokinase – EC: 2.7.1.2; *hk*, coding the hexokinase – EC: 2.7.1.1; *pfkl* and *pfkm* coding for the 6-phosphofructokinase – EC: 2.7.1.11, *pkl* and *pkm*, coding the pyruvate kinase – EC: 2.7.1.40) and gluconeogenesis (*pck* coding the phosphoenolpyruvate carboxykinase – EC: 4.1.1.32; *fbp* coding the fructose 1,6-bisphosphatase – EC: 3.1.3.11; *g6pc* coding the glucose 6-phosphatase – EC: 3.1.3.9) and lipogenesis (*g6pdh* coding the glucose 6-phosphate dehydrogenase – EC: 1.1.1.49; *acly* coding the adenosine triphosphate citrate lyase – EC: 2.3.3.8; *fas* coding the fatty acid synthase – EC: 2.3.1.85) and fatty acid metabolism [*hoad* coding the 3-hydroxyacyl-CoA dehydrogenase – EC: 1.1.1.35; *cpt1* coding the carnitine *O*-palmitoyltransferase 1 – EC: 2.3.1.21; *Δ6-fad* coding the acyl-CoA 6-desaturase (delta-6 desaturase) – EC:1.14.19.3; *elovl2* and *elovl5* coding the elongation of very long chain fatty acids protein – EC:2.3.1.199] and cholesterol metabolism (*hmgcs* coding the hydroxymethylglutaryl-CoA synthase – EC:2.3.3.10; *dhcr7* coding the 7-dehydrocholesterol reductase – EC:1.3.1.21; *cyp7a1* coding the cholesterol 7 alpha-monooxygenase – EC:1.14.14.23) and mitochondrial energy metabolism (*cs* coding the citrate synthase – EC:2.3.3.1; *sdhb* coding succinate dehydrogenase (ubiquinone) iron-sulfur subunit – EC:1.3.5.1).

**Table 2:** Primer sequences and accession numbers for qPCR analysis.

Metabolism Pathway	Gene name	Forward primer (5′-3′)	Reverse primer (5′-3′)	Accession Number (Genoscope, GenBank, Sigenae)
Glucose transporter	*glut2a*	GACAGGCACTCTAACCCTAG	CTTCCTGCGTCTCTGTACTG	GSONMG00024093001
	*glut2b*	CTATCAGAGAACGGTACAGGG	CAGGAAGGATGACACCACG	GSONMG00057853001
	*glut4a*	CATCTTTGCAGTGCTCCTTG	CAGCTCTGTACTCTGCTTGC	GSONMG00067238001
	*glut4b*	TCGGCTTTGGCTTCCAATATG	GTTTGCTGAAGGTGTTGGAG	GSONMG00016098001
Glycolysis	*gcka*	CTGCCCACCTACGTCTGT	GTCATGGCGTCCTCAGAGAT	GSONMG00033781001
(liver type)	*gckb*	TCTGTGCTAGAGACAGCCC	CATTTTGACGCTGGACTCCT	GSONMG00012878001
	*pfkla*	GATCCCTGCCACCATCAGTA	GTAACCACAGTAGCCTCCCA	GSONMG00009459001
	*pfklb*	AGTGCTCGCTGTAAGGTCTT	GTGATCCGGCCTTTCTGAAC	GSONMG00001975001
	*pkl*	CCATCGTCGCGGTAACAAGA	GCCCCTGGCCTTTCCTATGT	ContigAF246146.s.om.10
Gly colysis	*hk*	CTGGGACGCTGAAGACCAGA	CGGTGCTGCATACCTCCTTG	AY864082
(muscle type)	*pfkmaa*	GTCAGTCTGTCCGGTAACCA	ATCTGGAGGGTTGATGTGGG	GSONMG00028237001
	*pfkmab*	TCAGCGGAGGAGGCTAATC	GACTCTGTGCAGTAGTCGTG	GSONMG00035246001
	*pfkmba*	CTGGGCATGAAAAGGCGAT	GTCTTCTTGATGATGTGCTCCA	GSONMG00075887001
	*pfkmbb*	CGGTCGTATCTTTGCCAACATG	TGTCCATTTCCACAGTGTCATATT	GSONMG00069910001
	*pkmaa*	ACATTGCCCCCTACAGTTAC	AAGTGGAAATGAATGGGACGT	GSONMG00052888001
	*pkmab*	TGCTGAGGGCAGTGACGTA	AGCTCCTCAAACAGCTGTCTG	GSONMG00039304001
	*pkmba*	CAAGCCTGCCAACGATGTC	CAAGGAACAAGCACAACACG	GSONMG00032476001
	*pkmbb*	CAACTGTGACGAGAAGCACC	GAGCCCAGAGTACCACCATT	GSONMG00050270001
Gluconeogenesis	*pck1*	ACAGGGTGAGGCAGATGTAGG	CTAGTCTGTGGAGGTCTAAGGGC	GSONMG00082468001
	*pck2*	ACAATGAGATGATGTGACTGCA	TGCTCCATCACCTACAACCT	GSONMG00059643001
	*fbp1a*	GACAGAGGACGACCCGTG	GTACTGACCGGGTCCAACAT	GSONMT00001932001
	*fbp1b1*	CTCTCAAGAACCTCTACAGCCT	TCAGTTCTCCCGTTCCCTTC	GSONMT00063051001
	*fbp1b2*	ATCAGCAGGAATAGGTCGCG	CCTCCTCCAGCACGAATCTC	GSONMG00015701001
	*g6pca*	GATGGCTTGACGTTCTCCT	AGATCCAGGAGAGTCCTCC	GSONMG00076843001
	*g6pcb1a*	GCAAGGTCCAAAGATCAGGG	GCCAATGTGAGATGTGATGGG	GSONMG00076841001
	*g6pcb1b*	GCTACAGTGCTCTCCTTCTG	TCACCCCATAGCCCTGAAA	GSONMG00066036001
	*g6pcb2a*	ATCGGACAATACACACAGAACT	CAACTGATCTATAGCTGCTGCCT	GSONMG00013076001
	*g6pcb2b*	CCTCTGCTCTTCTGACGTAG	TGTCCATGGCTGCTCTCTAG	GSONMG00014864001
Lipogenesis	*g6pdh*	CTCATGGTCCTCAGGTTTG	AGAGAGCATCTGGAGCAAGT	GSONMG00076312001
	*acly*	GCTTTTGCCACGGTGGTCTC	GCTTCCGCTACGCCAATGTC	GSONMG00010247001
	*fas*	GTGATGTCGAGCTTCGTGCT	CTCCAGTGTCTGACGCACCT	tcaa0001c. m. 06_5. 1. om. 4
Fatty acid oxidation	*hoad*	GGACAAAGTGGCACCAGCAC	GGGACGGGGTTGAAGAAGTG	tcad0001a.i.15_3.1.om
	*cpt1b*	CCCTAAGCAAAAAGGGTCTTCA	CATGATGTCACTCCCGACAG	AF606076
Long chain PUFA biosynthesis	*Δ6-fad*	AGGGTGCCTCTGCTAACTGG	TGGTGTTGGTGATGGTAGGG	AF301910
	*elovl2*	TGTGGTTTCCCCGTTGGATGCC	ACAGAGTGGCCATGTCCTTGT	FYV3OTN01A4WMI.s.om.10
	*elovl5*	GAACAGCTTCATCCATGTCC	TGACTGCACATATCGTCTGG	AY605100
Cholesterol metabolism	*hmgcs*	AGTGGCAAAGAGAGGGTGT	TTGTGGTTGGAGACGAGGA	GSONMG00010243001
	*dhcr7*	GTAACCCACCAGACCCAAGA	CCTCTCCTATGCAGCCAAAC	GSONMG00025402001
	*cyp7a1*	ACGTCCGAGTGGCTAAAGAG	GGTCAAAGTGGAGCATCTGG	GSONMG00066448001
	*abcg5*	CACCGACATGGAGACAGAAA	GACAGATGGAAGGGGATGAA	GSONMG00075025001
	*abcg8*	GATACCAGGGTTCCAGAGCA	CCAGAAACAGAGGGACCAGA	GSONMG00075024001
	*abca1*	CAGGAAAGACGAGCACCTT	TCTGCCACCTCACACACACTTC	GSONMG00078741001
	*lxra*	TGCAGCAGCCGTATGTGGA	GCGGCGGGAGCTTCTTGTC	GSONMG00014026001
	*srebp-2*	TAGGCCCCAAAGGGATAAG	TCAGACACGACGAGCACAA	GSONMT00039651001
Mitochondrial metabolism	*cs*	GGCCAAGTACTGGGAGTTCA	CTCATGGTCACTGTGGATGG	–
	*qcr2*	CGTCACGCCTAGACTTCCTC	CTTCCAGGATCGGTGTGTTT	–
	*cox2*	CACTCCTGAGCCGTTCCTTC	GGGTACCGCTTCAACAACGA	–
	*cox4*	TACGTGGGGGACATGGTGTT	CCCAGGAGCCCTTCTCCTTC	–
	*atp5*	AGGTGGCTGGTACCATGAAG	TCTTGGAAGGGTCCATCTTG	–
	*sdhb*	CCCAGGATCAAGAAGTTCCA	TTAAGCAGGCCAGTGTGTTG	–
Reference gene	*ef1α*	TCCTCTTGGTCGTTTCGCTG	ACCCGAGGGACATCCTGTG	AF498320


***Glucose transporters:***glut2a and glut2b*, glucose transporter 2 paralogs*; glut4a and glut4b*, glucose transporter 4 paralogs. **Glycolysis in the liver:***gcka and gckb*, glucokinase paralogs; *pfkla and pfklb*, 6-phosphofructokinase, liver type, paralogs; *pkl*, pyruvate kinase, liver type. **Glycolysis in the muscle:***hk*, hexokinase; *pfkmaa, pfkmab, pfkmba* and *pfkmbb*, 6-phosphofructokinase, muscle type, paralogs; *pkmaa, pkmab, pkmba* and *pkmbb*, pyruvate kinase, muscle type, paralogs. **Gluconeogenesis:***pck1 and pck2*, phosphoenolpyruvate carboxykinase paralogs; *fbp1b1, fbp1b2 and fbp1a*, fructose 1,6-bisphosphatase paralogs; *g6pca, g6pcb1a, g6pcb1b*, *g6pcb2a* and g6pcb2b, glucose 6-phosphatase paralogs. **Lipogenesis:***g6pdh*, glucose 6-phosphate dehydrogenase; *acly*, adenosine triphosphate citrate lyase; *fas*, fatty acid synthase. **Fatty acid oxidation:***hoad*, 3-hydroxyacyl-CoA dehydrogenase; *cpt1b*, carnitine *O*-palmitoyltransferase 1B. **Long chain PUFA biosynthesis:***Δ6-fad*, acyl-CoA 6-desaturase (delta-6 desaturase)*; elovl2*, elongation of very long-chain fatty acid protein 2; *elovl5*, elongation of very long-chain fatty acid protein 5. **Cholesterol metabolism:***hmgcs*, hydroxymethylglutaryl-CoA synthase; *dhcr7*, 7-dehydrocholesterol reductase; *cyp7a1*, cholesterol 7 alpha-monooxygenase; *abcg5*, ATP-binding cassette transporter G5; *abcg8*, ATP-binding cassette transporter G8; *abca1*, ATP-binding cassette transporter A1; *lxra*, liver X receptor A. *srebp-2*, sterol regulatory element-binding protein 2. **Mitochondrial metabolism*:****cs*, citrate synthase; *qcr2*, ubiquitinol cytochrome c reductase core protein 2; *cox2 and cox4*, cytochrome C oxidase; *atp5a*, ATP synthase, H+ transporting, mitochondrial F1 complex, alpha subunit 1; *sdhb*, succinate dehydrogenase (ubiquinone) iron-sulfur subunit. **Reference gene:***ef1α*, elongation factor-1 alpha.*

Quantitative RT-PCRs were carried out on a Light Cycle 480 II (Roche Diagnostics, Neuilly-sur-Seine, France) using SYBR Green I Master (Roche Diagnostics GmbH, Mannheim, Germany). PCR were performed using 2 μl of the diluted cDNA (76 times) mixed with 0.24 μl of each primer (10 μM), 3 μl of Light Cycle 480 SYBR Green I Master (Roche Diagnostics) and 0.52 μl of DNase/RNase/protease-free water (5 prime, Hamburg, Germany) in a total volume of 6 μl. The qPCR were initiated at 95°C for 10 min, then followed by 45 cycles of a three-step amplification program (15 s at 95°C, 10 s at 60°C, 15 s at 72°C). Melting curves were systematically monitored (5 s at 95°C, 1 min at 65°C, temperature gradient 0.11°C/s from 65 to 97°C) at the end of the last amplification cycle to confirm the specificity of the amplification reaction. Each PCR assay included replicate samples (duplicate of reverse transcription and PCR amplification, respectively) and negative controls (reverse transcriptase and RNA free samples). Relative quantification of target genes expression was performed using the E-Method from the Light Cycler 480 software (version SW 1.5; Roche Diagnostics). PCR efficiencies were measured by the slope of a standard curve using serial dilution of cDNA (a pool of all cDNA samples), and they ranged between 1.8 and 2.0.

### Statistical Analysis

Normality of distributions was assessed by Shapiro–Wilk test. Data were analyzed by two-ways *ANOVA* to assess the differences between lines, diets and interactions. If interactions between diets and lines were statistically significant, a *post hoc* Tukey test was applied to compare all the groups. Analyses were performance with R software (v.3.3.3)/R Commander Package. Treatment effects and interactions were considered statistically significant at *p* < 0.05. Results were presented as means ± SD (*n* = 6 samples per group).

## Results

### Growth Performance and Whole-Body Composition

After 12 weeks, growth performance of the two isogenic fish fed with or without carbohydrate were obtained. Regarding the effect of the diet, feed intake (FI), specific growth rate (SGR), feed efficiency (FE), and protein retention efficiency (PRE) were significantly higher in trout fed H-CHO diet (Table 3, *p* < 0.05). A significant difference of FI was found between the two lines (*p* < 0.05), N38h showed higher FI than A22h (*p* < 0.05). There were no significant interactions of lines and diets for any these traits (*p* > 0.05).

**Table 3:** Growth performance and feed utilization in two isogenic lines of rainbow trout fed with low carbohydrate diet and high carbohydrate diet.

Line	A22h		N38h		*P*-value		
Diet	L-CHO	H-CHO	L-CHO	H-CHO	Line (L)	Diet (D)	L^∗^D
FI (g/d)	0.397 ± 0.03	0.490 ± 0.02	0.453 ± 0.02	0.540 ± 0.01	**0.001**	**3.0e^-5^**	0.8
SGR (%/d)	1.7 ± 0.06	2.2 ± 0.04	1.8 ± 0.06	2.2 ± 0.06	0.5	**7.7e^-7^**	0.5
FE	0.9 ± 0.03	1.2 ± 0.03	0.9 ± 0.03	1.1 ± 0.02	0.3	**2.5e^-7^**	0.7
PRE (%)	30.3 ± 1.5	37.3 ± 0.6	29.3 ± 0.6	37.3 ± 0.6	0.2	**4.9e^-7^**	0.5


*Data were presented as mean ± SD (*n* = 3 tanks), statistical differences were evaluated by Two-way *ANOVA* (*p* < 0.05, values in bold). Feed intake (FI, g/d) = (feed intake in dry matter, g)/days. Specific growth rate (SGR, % d-1) = 100 × [Ln (final body weight, g) - Ln (initial body weight, g)]/ days. Feed efficiency (FE) = (final-initial body weight, g)/(feed intake in dry matter, g). Protein retention efficiency (PRE, %) = 100 × (final-initial protein content, g)/(protein intake, g).*

Whole body compositions were shown in the Table 4. Whole body lipid and energy content were significantly higher in trout fed H-CHO diet than in those fed the L-CHO diet (*p* < 0.05). Only crude lipid showed difference between fish lines, A22h having a higher body lipid than N38h (*p* < 0.05). No significant difference of whole body protein content was found (*p* > 0.05).

**Table 4:** Whole body composition in two isogenic lines of rainbow trout (A22h and N38h) fed with low carbohydrate diet (L-CHO) and high carbohydrate diet (H-CHO).

Lines	A22h		N38h		*P*-Value		
Diets	L-CHO	H-CHO	L-CHO	H-CHO	Line (L)	Diet (D)	L^∗^D
Crude protein (%)	15.6 ± 0.3	15.5 ± 0.3	15.4 ± 0.2	15.5 ± 0.1	0.6	0.9	0.4
Crude lipid (%)	7.8 ± 0.3	9.4 ± 0.5	7.3 ± 0.2	9.0 ± 0.4	**0.04**	**3.4e^-5^**	0.8
Gross energy (kJ g^-1^)	6.8 ± 0.1	7.5 ± 0.2	6.6 ± 0.1	7.4 ± 0.2	0.09	**5.7e^-5^**	0.5


*Data were presented as mean ± SD (*n* = 6 fish), statistical differences were evaluated by Two-way *ANOVA* (*p* < 0.05, values in bold).*

### Postprandial Plasma Metabolites Levels

Plasma glucose, triglycerides and cholesterol were measured 6 h after the last meal (Table 5). Higher plasma glucose and triglycerides were observed in fish fed with H-CHO diet (*p* < 0.05) than in fish fed the L-CHO diet. Moreover, only plasma cholesterol was different bewteen trout lines, being higher in A22h (*p* < 0.05).

**Table 5:** Plasma metabolites level (g/L) in two isogenic lines of rainbow trout (A22h and N38h) fed with low carbohydrate diet (L-CHO) and high carbohydrate diet (H-CHO) 6 h after the last meal.

Lines	A22h		N38h		*P*-value		
Diets	L-CHO	H-CHO	L-CHO	H-CHO	Line (L)	Diet (D)	L^∗^D
Glucose (g/L)	0.8 ± 0.1	1.1 ± 0.5	0.9 ± 0.05	1.2 ± 0.2	0.3	**0.01**	0.9
Triglyceride (g/L)	5.0 ± 0.8	11.8 ± 3.5	5.0 ± 0.5	10.5 ± 2.4	0.5	**1.2e^-6^**	0.5
Cholesterol (g/L)	6.6 ± 0.7	6.3 ± 0.7	5.9 ± 0.3	5.7 ± 0.5	**0.007**	0.3	1.0


*Data were presented as mean ± SD (*n* = 6 fish), statistical differences were evaluated by Two-way *ANOVA* (*p* < 0.05, values in bold).*

### Metabolism in Liver

**FIGURE 1:**
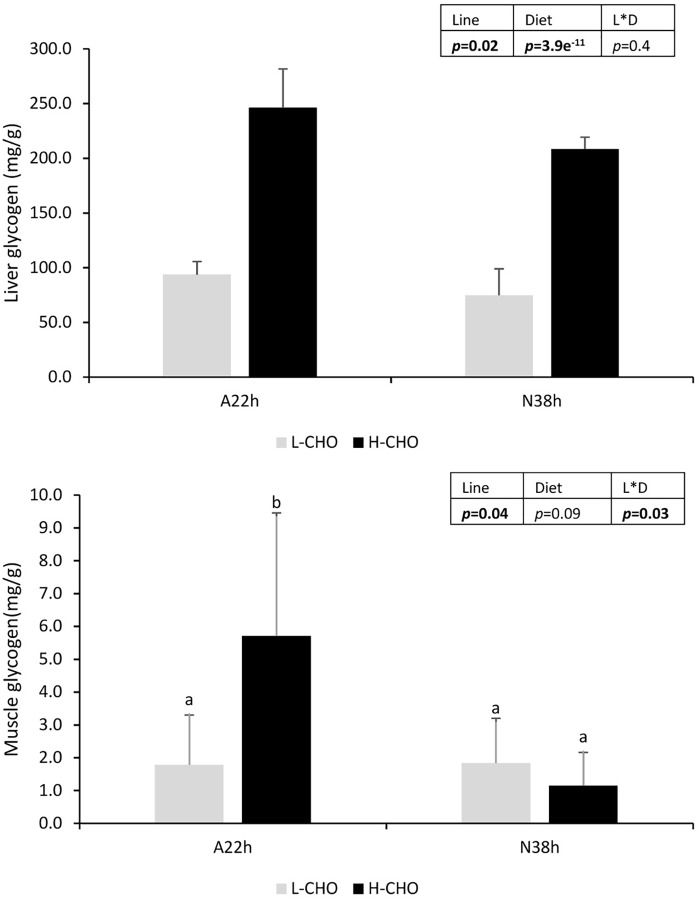
Liver and muscle glycogen in two isogenic lines of rainbow trout (A22h and N38h) fed with low carbohydrate diet (L-CHO) and high carbohydrate diet (H-CHO). Data were presented as mean ± SD (*n* = 6), statistical differences were evaluated by Two-way *ANOVA* (*p* < 0.05, values in bold). In case of interaction, *a post hoc* Tukey’s test was performed (*p* < 0.05, values in bold). Different superscripts indicated significant differences between treatments.

Liver glycogen contents was measured at 6 h after the last meal as shown in Figure 1. Liver glycogen was significantly increased in fish fed with H-CHO diet (*p* < 0.05), whereas A22h had higher hepatic glycogen content than N38h (*p* < 0.05).

We studied mRNA levels of glucose tranporter 2 genes. The higher expression of *glut2a* was found in trout fed H-CHO diet (Table 6, *p* < 0.05) whereas *glut2b* was higher expressed in A22h (Table 6, *p* < 0.05).

**Table 6:** mRNA levels of selected genes in the liver of two isogenic lines of rainbow trout (A22h and N38h) fed with low carbohydrate diet (L-CHO) and high carbohydrate diet (H-CHO) 6 h after the last meal.

Lines	A22h		N38h		*P*-value		
Diets	L-CHO	H-CHO	L-CHO	H-CHO	Line (L)	Diet (D)	L^∗^D
**Glucose transporter**				
*glut2a*	0.8 ± 0.1	1.0 ± 0.2	0.9 ± 0.2	1.1 ± 0.1	0.4	**0.02**	0.6
*glut2b*	1.2 ± 0.1	1.1 ± 0.2	1.0 ± 0.1	1.0 ± 0.2	**0.04**	1.0	0.5
**Glycolysis**				
*gcka*	0.01 ± 0.01^a^	2.3 ± 0.9^c^	0.1 ± 0.1^a^	1.2 ± 0.6^b^	**0.02**	**2.6e^-8^**	**0.01**
*gckb*	0.1 ± 0.1^a^	2.6 ± 0.8^c^	0.3 ± 0.2^a^	1.4 ± 0.5^b^	**0.02**	**2.2e^-8^**	**0.002**
*pfkla*	1.1 ± 0.2	0.8 ± 0.1	1.0 ± 0.2	0.6 ± 0.2	0.2	**2.8e^-4^**	0.3
*pfklb*	0.9 ± 0.2	0.7 ± 0.1	0.9 ± 0.1	0.6 ± 0.1	0.7	**0.002**	0.2
*Pkl*	1.6 ± 0.3	0.9 ± 0.2	1.6 ± 0.4	0.8 ± 0.1	0.3	**7.7e^-4^**	0.9
**Gluconeogenesis**				
*pck1*	1.3 ± 0.6	0.1 ± 0.04	1.2 ± 0.4	0.3 ± 0.1	0.7	**6.1e^-7^**	0.3
*pck2*	1.3 ± 0.2	1.2 ± 0.2	1.2 ± 0.3	1.2 ± 0.3	0.7	0.9	0.8
*fbp1b1*	1.0 ± 0.2	0.9 ± 0.2	0.9 ± 0.1	0.8 ± 0.1	0.1	0.07	0.6
*fbp1b2*	1.1 ± 0.2	0.8 ± 0.1	1.1 ± 0.1	0.8 ± 0.2	0.8	**0.003**	0.9
*fbp1a*	0.7 ± 0.4^a^	2.6 ± 1.1^b^	0.1 ± 0.04^a^	0.8 ± 0.4^a^	**1.5e^-4^**	**8.0e^-5^**	**0.02**
*g6pca*	0.6 ± 0.1	1.1 ± 0.7	0.6 ± 0.2	1.1 ± 0.3	1.0	**0.004**	1.0
*g6pcb1a*	0.9 ± 0.4	1.2 ± 0.3	0.9 ± 0.1	1.2 ± 0.2	0.7	**0.05**	0.9
*g6pcb1b*	0.7 ± 0.4	0.7 ± 0.5	0.9 ± 0.4	1.1 ± 0.4	0.09	0.4	0.9
*g6pcb2a*	0.7 ± 0.3^a^	2.5 ± 0.9^b^	0.1 ± 0.04^a^	0.7 ± 0.5^a^	**5.8e^-5^**	**3.2e^-5^**	**0.01**
**Fatty acid metabolism**				
*g6pdh*	0.3 ± 0.1	1.7 ± 0.9	0.3 ± 0.05	1.6 ± 0.4	0.9	**2.4e^-7^**	0.9
*acly*	0.5 ± 0.1	1.6 ± 0.3	0.5 ± 0.1	1.6 ± 0.6	0.9	**1.0e^-6^**	1.0
*fas*	0.3 ± 0.2	2.2 ± 0.3	0.4 ± 0.1	1.5 ± 0.8	0.2	**1.1e^-6^**	0.1
*hoad*	0.7 ± 0.1	1.4 ± 0.1	0.6 ± 0.2	1.6 ± 0.	0.9	**6.8e^-8^**	0.3
*Δ6-fad*	1.6 ± 0.3	1.5 ± 0.7	0.8 ± 0.3	1.2 ± 0.4	**0.004**	0.4	0.2
*elovl2*	1.9 ± 0.4	1.9 ± 0.8	0.6 ± 0.	0.9 ± 0.3	**3.5e^-5^**	0.4	0.5
*elovl5*	1.6 ± 0.3	1.1 ± 0.3	1.0 ± 0.4	0.8 ± 0.2	**0.001**	**0.01**	0.3
**Cholesterol metabolism**				
*hmgcs*	0.2 ± 0.2	1.4 ± 0.4	0.1 ± 0.1	1.0 ± 1.0	0.3	**2.2e^-4^**	0.4
*dhcr7*	0.3 ± 0.1	0.9 ± 0.4	0.3 ± 0.	1.2 ± 0.4	0.3	**7.2e^-6^**	0.2
*cyp7a1*	0.9 ± 0.2	0.8 ± 0.3	0.9 ± 0.2	1.1 ± 0.3	0.1	0.7	0.1
*abcg5*	0.8 ± 0.2	1.3 ± 0.3	0.7 ± 0.4	0.9 ± 0.4	0.08	**0.01**	0.3
*abcg8*	0.7 ± 0.2	0.8 ± 0.2	1.2 ± 0.2	1.1 ± 0.3	**0.0003**	1.0	0.8
*abca1*	0.9 ± 0.3	0.8 ± 0.2	1.0 ± 0.2	0.8 ± 0.3	0.5	0.09	0.3
*lxra*	0.9 ± 0.2	0.8 ± 0.3	1.1 ± 0.2	0.9 ± 0.2	0.09	0.3	0.8
*srebp-2*	0.8 ± 0.4	1.2 ± 0.	0.7 ± 0.2	0.6 ± 0.2	**0.02**	0.2	0.1
**Mitochondrial energy metabolism)**				
*cs*	0.9 ± 0.2	1.0 ± 0.	0.9 ± 0.2	1.3 ± 0.3	0.2	**0.03**	0.2
*qcr2*	0.9 ± 0.2	1.0 ± 0.4	0.8 ± 0.2	1.2 ± 0.2	1.0	**0.04**	0.2
*cox2*	0.8 ± 0.1	0.8 ± 0.2	1.0 ± 0.2	0.8 ± 0.3	0.2	0.6	0.4
*cox4*	0.7 ± 0.1	1.0 ± 0.4	0.9 ± 0.2	1.2 ± 0.2	0.05	**0.01**	0.4
*atp5a*	0.9 ± 0.2	1.1 ± 0.4	0.7 ± 0.1	1.2 ± 0.2	1.0	**0.03**	0.3
*sdhb*	0.8 ± 0.2	0.9 ± 0.3	0.7 ± 0.2	1.1 ± 0.5	0.4	0.09	0.4


*Data were presented as mean ± SD (*n* = 6 fish), statistical differences were evaluated by Two-way *ANOVA* (*p* < 0.05, values in bold). In case of interaction, *a post-hoc* Tukey’s test was performed (*p* < 0.05, values in bold). Different superscripts indicated significant differences between groups. *glut2a and glut2b*: glucose transporter 2 paralogs. *gcka and gckb*: glucokinase paralogs. *pfkla* and *pfklb:* 6-phosphofructokinase, liver type, paralogs. *pkl:* pyruvate kinase, liver type. *pck1* and *pck2*: phosphoenolpyruvate carboxykinase paralogs. *fbp1b1, fbp1b2 and fbp1a*: fructose 1,6-bisphosphatase paralogs. *g6pca, g6pcb1a, g6pcb1b and g6pcb2a*: glucose 6-phosphatase paralogs. *g6pcdh*: glucose 6-phosphate dehydrogenase. *acly*: adenosine triphosphate citrate lyase. *fas*: fatty acid synthase. *hoad*: 3-hydroxyacyl-CoA dehydrogenase. *Δ6-fad*: acyl-CoA 6-desaturase (delta-6 desaturase). *elovl2:* elongation of very long-chain fatty acid protein 2. *elovl5:* elongation of very long-chain fatty acid protein 5. *hmgcs*: hydroxymethylglutaryl-CoA synthase. *dhcr7:* 7-dehydrocholesterol reductase. *cyp7a1:* cholesterol 7 alpha-monooxygenase. *abcg5*: ATP-binding cassette transporter G5. *abcg8*: ATP-binding cassette transporter G8. *abca1*: ATP-binding cassette transporter A1. *lxra*: liver X receptor A. srebp-2: sterol regulatory element-binding protein 2. *cs*: citrate synthase. *qcr2*: ubiquitinol cytochrome c reductase core protein 2. *cox2 and cox4:* cytochrome C oxidase. *atp5a*: ATP synthase, H ^+^ transporting, mitochondrial F1 complex, alpha subunit 1. *sdhb:* succinate dehydrogenase (ubiquinone) iron-sulfur subunit.*

Regarding the glucose metabolism in liver, mRNA levels of target genes encoding key enzymes involved in glycolysis were analyzed (Table 6). Line^∗^diet interactions were found in the mRNA levels of *gcka* and *gckb*: A22h fed H-CHO diet showed significant higher expression of *gcka* and *gckb*, then followed by N38h fed H-CHO diet, both A22h and N38h fed L-CHO diet showed significant lower mRNA levels of *gcka* and *gckb* (*p* < 0.05). Moreoever, higher *pfkla, pfklb* and *pkl* mRNA levels were observed in trout fed with L-CHO diet. We also studied the mRNA levels of key gluconeogenic enzymes (Table 6). When fed with H-CHO diet, trout exhibited a significant decrease in the mRNA levels of *pck1* and *fbp1b2* but a significant increase in the mRNA levels of *g6pca* and *g6pcb1a* (*p* < 0.05). There were significant line^∗^diet interactions in the mRNA levels of *fbp1a* and *g6pcb2a*, an increased mRNA level of *fbp1a* and *g6pcb2a* were observed in A22h fed with H-CHO diet (*p* < 0.05).

We further analyzed lipid metabolism in liver. We measured the mRNA levels of selected enzymes involved in fatty acid metabolism and cholesterol metabolism in the liver (Table 6). H-CHO diet was associated with increased mRNA levels of *g6pdh*, *acly*, *fas* (all of them involved in lipogenesis), and also increased mRNA levels for *hoad* (one of the key enzyme of the*β*-oxidation). A22h showed significant higher mRNA levels of *Δ6-fad*, *elovl2* and *elovl5* involved in the LC-PUFA biosynthesis than N38h (*p* < 0.05). Moreover, *elovl5* is higher expressed in fish fed L-CHO diet (*p* < 0.05). Regarding the cholesterol metabolism, we found that *hmgcs*, *dhcr7* and *abcg5* were expressed at higher level in fish fed carbohydrate diet, whereas mRNA levels of *abcg8* and *srebp-2* were expressed at a higher level in A22h irrespective of the diets.

We also analyzed mitochondrial energy metabolism in trout liver as shown in Table 6. *cs*, *qcr2*, *cox4* and *atp5a* mRNA levels were higher in fish fed high carbohydrate (*p* < 0.05), but no differences were detected between the two lines (*p* > 0.05).

### Metabolism in Muscle

We also studied the gene expression in the muscle (Table 7). *glut4* was the main glucose tranporter form in the muscle. No difference in the expression of *glut4a* was found between lines and diets (*p* > 0.05). By contrast, *glut4b* was expressed at higher level in A22h (*p* < 0.05). On the other hand, there was a strong interaction between lines and diets for the muscle glycogen, because only A22h fed with H-CHO diet had significant higher muscle glycogen content (Figure 1, *p* < 0.05).

**Table 7:** mRNA levels of selected genes in the muscle of two isogenic lines of rainbow trout (A22h and N38h) fed with low carbohydrate diet (L-CHO) and high carbohydrate diet (H-CHO) 6h after the last meal.

Lines	A22h		N38h		*P*-value		
Diets	L-CHO	H-CHO	L-CHO	H-CHO	Line (L)	Diet (D)	L^∗^D
**Glucose transporter**			
*glut4a*	1.0 ± 0.6	1.0 ± 0.4	0.8 ± 0.1	0.9 ± 0.5	0.5	0.8	0.6
*glut4b*	1.1 ± 0.4	0.8 ± 0.1	1.1 ± 0.2	1.4 ± 0.4	**0.01**	1.0	0.05
**Glycolysis**			
*hk*	0.9 ± 0.4	0.8 ± 0.1	0.8 ± 0.1	0.8 ± 0.1	0.4	1.0	0.4
*pfkmaa*	1.0 ± 0.4	1.1 ± 0.2	1.0 ± 0.3	1.1 ± 0.2	0.8	0.6	0.7
*pfkmab*	1.0 ± 0.3	1.3 ± 0.2	1.0 ± 0.3	1.4 ± 0.3	0.8	**0.01**	0.7
*pfkmba*	0.4 ± 0.2	1.3 ± 0.3	0.8 ± 0.3	1.6 ± 0.30	**0.003**	**4.6e^-7^**	0.8
*pfkmbb*	0.9 ± 0.3	1.2 ± 0.3	0.9 ± 0.2	1.2 ± 0.2	0.8	**0.009**	0.9
*pkmaa*	0.8 ± 0.3	0.8 ± 0.2	1.1 ± 0.5	1.4 ± 0.3	**0.006**	0.3	0.3
*pkmab*	1.2 ± 0.4	1.6 ± 0.2	0.9 ± 0.2	1.5 ± 0.4	0.3	**0.02**	0.3
*pkmba*	0.6 ± 0.3	1.1 ± 0.2	0.9 ± 0.2	1.3 ± 0.2	**0.01**	**2.3e^-4^**	1.0
*pkmbb*	0.7 ± 0.3	1.1 ± 0.2	0.9 ± 0.2	1.3 ± 0.3	**0.03**	**8.4e^-4^**	0.8
**Fatty acid oxidation**			
*hoad*	1.0 ± 0.3	0.9 ± 0.1	1.0 ± 0.2	1.0 ± 0.3	0.6	0.9	0.5
*cpt1b*	0.5 ± 0.1	0.4 ± 0.1	0.6 ± 0.1	0.8 ± 0.1	**0.006**	0.2	0.2
**Mitochondrial energy metabolism**			
*cs*	1.0 ± 0.4	0.8 ± 0.1	1.0 ± 0.2	1.1 ± 0.2	0.1	0.3	0.1
*qcr2*	0.9 ± 0.3	0.9 ± 0.1	1.0 ± 0.1	1.0 ± 0.1	0.2	0.9	0.9
*cox2*	1.1 ± 0.4	0.7 ± 0.04	1.0 ± 0.3	0.9 ± 0.3	0.9	**0.04**	0.1
*cox4*	0.9 ± 0.2	1.0 ± 0.2	1.0 ± 0.1	1.1 ± 0.2	0.4	0.1	0.8
*atp5a*	1.0 ± 0.4	1.0 ± 0.1	1.0 ± 0.2	1.0 ± 0.2	1.0	0.9	0.6
*sdhb*	1.1 ± 0.4	0.7 ± 0.1	1.0 ± 0.2	0.9 ± 0.2	0.4	**0.008**	0.09


*Data were presented as mean ± SD (*n* = 6 fish), statistical differences were evaluated by Two-way *ANOVA* (*p* < 0.05, values in bold). *glut4a and glut4b*: glucose transporter 4 paralogs. *hk*: hexokinase. *pfkmaa, pfkmab, pfkmba* and *pfkmbb:* 6-phosphofructokinase, muscle type, paralogs. *pkmaa, pkmab, pkmba* and *pkmbb:* pyruvate kinase, muscle type, paralogs. *hoad*: 3-hydroxyacyl-CoA dehydrogenase. *cpt1b:* carnitine *O*-palmitoyltransferase 1B. *cs*: citrate synthase. *qcr2*: ubiquitinol cytochrome c reductase core protein 2. *cox2 and cox4:* cytochrome C oxidase. *atp5a*: ATP synthase, H^+^ transporting, mitochondrial F1 complex, alpha subunit 1. *sdhb:* succinate dehydrogenase (ubiquinone) iron-sulfur subunit.*

Although no significant difference of *hk* mRNA level was observed between lines nor diets (*p* > 0.05), most genes involved in the glycolysis were up-regulated by carbohydrate enriched diet. Indeed, the mRNA levels of *pfkmab*, *pfkmba*, *pfkmbb*, *pkmab*, *pkmba*, and *pkmbb* were all higher in trout muscle when fish were fed with H-CHO diet (*p* < 0.05). Moreover, some genes such as *pfkmba*, *pkmaa*, *pkmba* and *pkmbb* were higher expressed in N38h than in A22h (*p* < 0.05).

Regarding fatty acid metabolism in the muscle, we found that *cpt1b* invloved in *β*-oxidation was higher expressed in N38h than in A22h (*p* < 0.05).

We also analyzed mitochondrial energy metabolism in the muscle of the two lines. Only a few differences were observed, *cox2* and *sdhb* mRNA levels were lower in fish fed H-CHO diet (*p* < 0.05).

## Discussion

Rainbow trout is considered as a poor user of dietary carbohydrate as reflected by the persistent postprandial hyperglycemia after feeding high levels of carbohydrates or after a glucose challenge tolerance test. Although several studies explored some hypotheses to explain its poor utilization (see reviews by Polakof et al., 2012; Kamalam et al., 2017), the underlying regulations of metabolic pathways (not only glucose metabolism) are not fully understood. Some questions still need to be explored: (i) could dietary carbohydrate be a regulator of glucose metabolism and other metabolic pathways in trout? (ii) is the atypical regulation of glucose metabolism observed previously with two isogenic trout lines a general feature? Thus, we compared two new isogenic lines A22h and N38h fed with (23%) or without (3.6%) digestible carbohydrates to answer these two questions.

### Dietary Carbohydrate Is Associated With Higher Growth and Feed Efficiency (Due to Protein-Sparing Effect) and Affects Intermediary Metabolism in Liver and Muscle of Rainbow Trout

#### Dietary Carbohydrates, Growth Performance and Glycemia

Dietary carbohydrate is dispensable for fish (The National Research Council [NRC], 2011). By contrast, very high level of inclusion of dietary carbohydrates decreases growth performance and induced metabolic disorder in carnivorous fish (Bergot, 1979; Enes et al., 2009; Skiba-Cassy et al., 2013; Kamalam et al., 2017). In the present experiment, no negative effects of H-CHO diet on the growth of trout, instead, an improvement of SGR was induced by H-CHO diet. It was consistent with what had been previously reported, to some extent, appropriate supplement of dietary carbohydrate could increase fish growth performance (Singh et al., 2006; Mohanta et al., 2007). Moreover, H-CHO diet improved FE and PRE, our data suggest the better feed utilization reflecting the well-known protein-sparing effect of digestible carbohydrates as described by Kaushik et al. (1989), Kim and Kaushik (1992), and Wilson (1994).

Post-prandial glycemia is a basic indicator for assessing glucose homeostasis after feeding with carbohydrate diet (Polakof et al., 2012; Kamalam et al., 2017). In the present study, an expected increase of the plasma glucose (6 h postprandial) was found in trout fed with H-CHO diet, but the glycemia was relatively low (1.1 – 1.2 g/l) compared to that in previous studies (Wilson, 1994; Hemre et al., 2002; Panserat et al., 2009). It may be due to the low content of lipid in the diets (8–11%), as we know that a low level of dietary lipids is associated with a better glycemia homoeostasis in trout fed with carbohydrates (Figueiredo-Silva et al., 2012). Besides, it could be due to an adaptative process because the experimental fish had been fed for a long period. Furthermore, we cannot also exclude that the isogenic lines represented specific genetic background associated to a particular use of nutrients.

#### Molecular Regulation by Dietary Carbohydrates of Intermediary Metabolism in Liver

One of the main objectives in the present study was to test the effects of feeding high vs. very low carbohydrate on hepatic intermediary metabolism, liver being the key organ for the regulation of metabolic homeostasis (Pilkis and Granner, 1992; Klover and Mooney, 2004). We observed a higher *glut2a* mRNA level in fish fed H-CHO diet; this was unexpected because this gene was not reported to be affected by dietary glucose (Kirchner et al., 2008; Polakof et al., 2010; Jin et al., 2014). As we already observed such an atypical regulation by dietary carbohydrates of hepatic glycolysis and gluconeogenesis as seen before in the two other isogenetic lines (A32h and AB1h) (Song et al., 2018) suggesting that it is likely a general rule in rainbow trout whatever the genetic background (i) although *gck (gcka and gckb)* were markedly induced by H-CHO diet, *pfkla, pfklb* and *pkl* genes encoding for the last two glycolytic enzymes were surprisingly lower expressed; (ii) only the two gluconeogenic genes (*pck1* and *fbp1b1*) were suppressed as expected, the other ones such as *fbp1a*, *g6pca*, *g6pcb1a* and *g6pcb2a* were all higher expressed in fish fed H-CHO diet. These data reinforced the hypothesis that an absence of concomitant increase of glycolysis and inhibition of gluconeogenesis in liver may contribute the establishment of the glucose-intolerant phenotype in rainbow trout (Enes et al., 2009; Marandel et al., 2015, 2016; Song et al., 2018).

We also analyzed some genes involved in hepatic lipid metabolism. Genes involved in lipogenesis (*g6pdh*, *acly* and *fas*) were up-regulated in fish fed H-CHO diet. Indeed, it was expected that H-CHO diet was associated with higher capacity of fatty acid synthesis and higher levels of plasma triglycerides and lipid content in whole body as previously observed (Skiba-Cassy et al., 2009; Jin et al., 2014). The increased mRNA level of *hoad* involved in the lipid β-oxidation in fish fed H-CHO diet was more unexpected. As for those of lipogenesis, the genes involved in the synthesis of cholesterol (*hmgcs* and *dhcr7*) and direct elimination of hepatic cholesterol into the bile (*abcg5*) were up-regulated in trout fed H-CHO diet. This is the demonstration for the first time of an effect of dietary carbohydrate on cholesterol metabolism at a molecular level in fish. Our results confirmed previous data suggesting an effect of dietary carbohydrates on cholesterol levels in fish (trout, seabass) fed with plant-based diets (Kamalam et al., 2013; Castro et al., 2015). As no difference of plasma cholesterol was noticed between diets, our results suggested that cholesterol synthesis may have been compensated by cholesterol elimination into the bile, thus maintaining cholesterol homeostasis.

Finally, regarding mitochondrial metabolism in the liver, the mRNA levels of many of the genes involved in energy production in mitochondria (*cs*, *qcr2*, *cox4*, and *atp5a*) were expressed at higher level in fish fed the H-CHO diet. These data previously observed can be linked directly to the dietary carbohydrates, but they are more probably related to the fact that the H-CHO diet is richer in digestible energy than the L-CHO diet (cellulose is not highly digestible in trout), and thus stimulated energy metabolism in high carbohydrate treatment.

#### Molecular Regulation by Dietary Carbohydrates of Intermediary Metabolism in Muscle

Muscle is the largest tissue of fish and may play a major role in the efficiency of nutrients utilizations. In the present study, we analyzed the main catabolic pathways including glycolysis, β-oxidation and miochondrial energy production in the muscle. Previous experiments revolved a low capacity of glucose utilization in fish muscle because of low efficiency of glucose uptake by muscle through glucose transporter (*glut4*, muscle type) and poor activation of HK and/or PK activities when the fish were fed carbohydrate enriched diet (Hemre et al., 2002; Panserat et al., 2009). In our study, no significant differences were observed for the mRNA levels of *glut4* (*glut4a* and *glut4b*) and *hk* in muscle confirming the previous observations (Kirchner et al., 2005; Díaz et al., 2007). However, glycolytic *pfkm* (including paralogs *pfkmaa*, *pfkmab*, *pfkmba*, *pfkmbb*) and *pkm* (including paralogs *pkmaa*, *pkmab*, *pkmba*, *pkmbb*) mRNA levels were higher in trout fed H-CHO diet, suggesting an adaptation to dietary carbohydrates in muscle for some of the glycolytic enzymes. By contrast, and not surprisingly because the two diets were designed isolipidic, we did not find any differences in the mRNA levels of *hoad* and *cpt1b* involved in fatty acid β-oxidation. Finally, and surprisingly, some genes (*cox2* and *sdhb*) involved in mitochondrial energy metabolism were expressed at lower level in fish fed H-CHO diet (with higher digestible energy) in the muscle. There were no clear reasons for these results which differ from those observed in the liver. Further studies are therefore needed to better understand this observation.

### Evidence for a Genetic Variability Are for Growth Performance and Intermediary Metabolism

Another important objective of our study was to investigate if the differences may occur between the two isogenic rainbow trout (A22h and N38h) lines when fed a diet with or without carbohydrate. In the experiment, although N38h had higher FI than A22h, no significant differences were found for growth performance and feed utilization between the two lines, thus we cannot demonstrate which genotype was advanced in carbohydrate utilization at least at the phenotype level.

Moreover, there were no significant differences in postprandial glycemia, but hepatic glycogen storage was higher in A22h than in N38h consistent with higher mRNA levels of *gcka* and *gckb* in A22h line, which could suggest a better storage of glucose through glycogen from A22h line. However, the *fbp1a* and *g6pcb2a* mRNA levels were also higher in A22h suggesting a poor control of the endogenous glucose production in this line. On the other hand, the mRNA levels of glucose transporter (*glut4b*) and glycolytic enzymes (*pfkmba*, *pkmaa*, *pkmba*, and *pkmbb*) in muscle were both lower in A22h. Therefore, there is no clear conclusion about a better regulation of glucose metabolism by carbohydrates by one of the two isogenic lines, as in previous comparison between two other isogenic lines (A32h and AB1h) (Song et al., 2018), suggesting the absence of polymorphism in glucose metabolism. Regarding the lipid metabolism and mitochondrial metabolism, differences between the two isogenic lines for genes involved in *β*-oxidation and mitochondrial metabolism used in the study were limited, except for the LC-PUFA biosynthesis capacity and cholesterol metabolism: in liver, higher mRNA levels of *Δ6-fad*, *elovl2* and *elovl5* and lower *abcg8* – in charge of hepatic cholesterol transport into the bile were detected in A22h, suggesting the existence of genetic variability in the control of lipid metabolism as reflected by the clear differences in plasma cholesterol.

## Conclusion

In conclusion, the present study described for the first time the effects of dietary carbohydrate on glucose, lipid and mitochondrial metabolism in the liver and muscle in two isogenic trout lines. These data confirmed the atypical regulation by dietary carbohydrates of hepatic glycolysis and gluconeogenesis (see Song et al., 2018 with two other studied lines) and the absence of regulation for muscle glucose transport and phosphorylation (Polakof et al., 2012). For the first time, the higher mRNA levels for genes involved in hepatic lipogenesis, cholesterol metabolism and energy metabolism as well as for those involved in glycolysis in the muscle by feeding carbohydrate were clearly demonstrated. Results also evidenced the existence of genetic variability for the expression of metabolic genes, especially for biosynthesis of EPA and DHA. Globally the present findings apport new data about the effects of dietary carbohydrates on intermediary metabolism and on the genetic control of this metabolism in rainbow trout. However, the present study on metabolic regulations was conducted only at the molecular level, and there was no data about at enzymatic/proteic levels which could corroborate the mRNA results. Thus, more integrated researches are needed in the future.

## Author Contributions

SP and IG designed the study. EQ and MD-N provided the isogenic lines. GC and SS-C provided the primers of cholesterol metabolism. LM provided the primers involved in glucose metabolism. XS performed the experiments and wrote the paper. LM, SS-C, GC, MD-N, EQ, IG, and SP gave suggestions about paper writing.

## Conflict of Interest Statement

The authors declare that the research was conducted in the absence of any commercial or financial relationships that could be construed as a potential conflict of interest.

## Footnotes

1 http://zfin.org/

## Abbreviations

*Δ6-fad*: acyl-CoA 6-desaturase (delta-6 desaturase)
*abca1*: ATP-binding cassette transporter A1
*abcg5*: ATP-binding cassette transporter G5
*abcg8*: ATP-binding cassette transporter G8
*acly*: adenosine triphosphate citrate lyase
*atp5a*: ATP synthase, H^+^ transporting, mitochondrial F1 complex, alpha subunit 1
*cox2 and cox4*: cytochrome C oxidase
*cpt1a*: carnitine *O*-palmitoyltrasferase 1A
*cpt1b*: carnitine *O*-palmitoyltrasferase 1B
*cs*: citrate synthase
*cyp7a1*: cholesterol 7alpha-monooxygenase
*dhcr7*: 7-dehydrocholesterol reductase
*ef1α*: elongation factor-1 alpha
*elovl2*: elongation of very long-chain fatty acid protein 2
*elovl5*: elongation of very long-chain fatty acid protein 5
*fas*: fatty acid synthase
*fbp1b1*, *fbp1b2* and *fbp1a*,: fructose 1,6-bisphosphatase, paralogs
*g6pca, g6pcb1a, g6pcb1b, g6pcb2a* and *g6pcb2b*: glucose 6-phosphatase, paralogs
*g6pdh*: glucose 6-phosphate dehydrogenase
*gcka and gckb*: glucokinase, paralogs
*glut2a and glut2b*: glucose transporter type 2, paralogs
*glut4a and glut4b*: glucose transporter type 4, paralogs
H-CHO: high carbohydrate diet
*hk*: hexokinase
*hmgcs*: hydroxymethylglutaryl-CoA synthase
*hoad*: 3-hydroxyacyl-CoA dehydrogenase
L-CHO: low carbohydrate diet
*lxra*: liver X receptor A
*pck1, pck2*: phosphoenolpyruvate carboxykinase, paralogs
*pfkla, pfklb*: 6-phosphofructokinase, liver type, paralogs
*pfkmaa, pfkmab, pfkmba and pfkmbb*: 6-phosphofructokinase, muscle type, paralogs
*pklr*: pyruvate kinase, liver type
*pkmaa, pkmab, pkmba and pkmbb*: pyruvate kinase, muscle type, paralogs
*qcr2*: ubiquitinol cytochrome c reductase core protein 2
*sdhb*: succinate dehydrogenase (ubiquinone) iron-sulfur subunit
*srebp-2*: sterol regulatory element-binding protein 2
